# FcγR-Mediated Trogocytosis 2.0: Revisiting History Gives Rise to a Unifying Hypothesis

**DOI:** 10.3390/antib11030045

**Published:** 2022-07-05

**Authors:** Margaret A. Lindorfer, Ronald P. Taylor

**Affiliations:** School of Medicine, University of Virginia, Charlottesville, VA 22908, USA; rpt@virginia.edu

**Keywords:** trogocytosis, Fcγ receptors, liver sinusoidal endothelial cells, immune adherence

## Abstract

There is increasing interest in the clinical implications and immunology of trogocytosis, a process in which the receptors on acceptor cells remove and internalize cognate ligands from donor cells. We have reported that this phenomenon occurs in cancer immunotherapy, in which cells that express FcγR remove and internalize CD20 and bound mAbs from malignant B cells. This process can be generalized to include other reactions including the immune adherence phenomenon and antibody-induced immunosuppression. We discuss in detail FcγR-mediated trogocytosis and the evidence supporting a proposed predominant role for liver sinusoidal endothelial cells via the action of the inhibitory receptor FcγRIIb2. We describe experiments to test the validity of this hypothesis. The elucidation of the details of FcγR-mediated trogocytosis has the potential to allow for the development of novel therapies that can potentially block or enhance this reaction, depending upon whether the process leads to unfavorable or positive biological effects.

## 1. Introduction

Almost 20 years ago, Joly and Hudrisier briefly reviewed trogocytosis [1]. This is a process in which antigen receptors on acceptor cells such as T lymphocytes form immunological synapses with antigen-presenting donor cells such as dendritic cells, and then fragments of membrane and associated antigen are transferred to the acceptor lymphocytes. Both donor and acceptor cells remain viable after this transfer reaction. They called this process trogocytosis based on the Greek term trogo, meaning gnaw. At that time, it was already known that T cells, B cells, and NK cells could mediate trogocytosis, and Joly and Hudrisier recognized that the process could be responsible for a wide variety of downstream immunological consequences. Their communication has been cited in more than 300 publications, clearly demonstrating the immunological importance of trogocytosis. In the past 5 years, the general subject of trogocytosis has been reviewed extensively [2,3,4,5,6,7,8,9,10] and recent evidence indicates that trogocytosis impacts a number of immunologic phenomena including the action of CAR-T cells and checkpoint inhibitors [11,12,13,14]. We will focus this review on one subclass of trogocytosis that is mediated by the recognition of IgG immune complexes on donor cells by FcγR on acceptor cells. The acceptor cells form immunological synapses with the IgG-opsonized cells, thus allowing for transfer of cell-bound IgG and associated antigen and membrane to the effector cells. 

Indeed, Nelson’s description of the immune adherence phenomenon, first reported almost 70 years ago, sets the stage for our review and evaluation of the mechanisms of trogocytosis mediated by FcγR [15,16]. His work demonstrated that bacteria opsonized with antibodies could activate complement, thus allowing for covalent tagging of this immune complex by activated complement proteins, later identified as multiple copies of C3b [17]. The C3b-tagged immune complex could then be immobilized on the surface of erythrocytes (E) via multivalent high avidity binding to clusters of complement receptor 1 (CR1), an E receptor that recognizes C3b [17,18]. These E-bound immune complexes could then be rapidly transferred to acceptor neutrophils and macrophages based on another multivalent interaction with several activating FcγR on these acceptor cells. In the absence of E, the direct uptake of the antibody-opsonized bacteria by the acceptor cells was quite weak, thus validating the efficacy and immunologic utility of immune adherence. Moreover, during this transfer process, the E are not phagocytosed or destroyed, and instead, as we have demonstrated in several model systems, CR1 and the associated chelated immune complexes are transferred to the acceptor macrophages [18]. Thus, we suggest that the immune adherence phenomenon is the first description of FcγR-mediated trogocytosis.

We will review compelling evidence which underlines the potential immunological implications and clinical importance of FcγR-mediated trogocytosis that has now emerged from in vitro investigations as well as from clinical studies and mouse models. These reports describe very similar reaction patterns for a number of additional antibody/donor cell systems, as follows: anti-E antibodies; Epstein–Barr virus (EBV)-immune complexes bound to B cells; T cells opsonized with anti-HIV antibodies; E and multiple myeloma (MM) cells opsonized with anti-CD38 mAb; and scenarios in which NK cells can act as either donors or acceptors during trogocytosis. In most cases, these trogocytosis reactions lead to decreases in the efficacies of mAb-based cancer therapies. Therefore, an enhanced understanding of the mechanistic details of these reactions should allow for the development of strategies to selectively block FcγR-mediated trogocytosis and enhance the therapies. 

Our in vitro studies of mAb-opsonized cancer cells indicated that immune effector cells, such as monocyte/macrophages, neutrophils, and NK cells, should play a leading role in vivo in FcγR mediated trogocytosis of these cells. Inherent in this concept was the idea that, in analogy to phagocytosis, it was the activating receptors on these cells (FcγRI, FcγRIIa, and FcγRIII) that promoted trogocytosis [19]. However, we strongly suggest that a paradigm shift is now in order; based on a considerable literature, we hypothesize that it is the inhibitory receptor FcγRIIb2 on liver sinusoidal endothelial cells (LSEC) that in many cases mediates trogocytosis in vivo by removing and clearing the small immune complexes displayed on IgG-opsonized circulating cells [20,21,22,23,24,25,26,27,28,29,30,31,32,33]. We will describe previous studies of the action of LSEC and immune complex clearance that support our hypothesis and based on the model systems that have already been investigated, we will delineate key new experiments that will allow for its rigorous testing. If these tests can validate the proposed mechanism, then downstream there are several focused strategies that can be evaluated to block the action of LSEC and thereby inhibit the negative effects of trogocytosis in several mAb-based therapies. 

## 2. Generalization of the Process 

**Figure 1:**
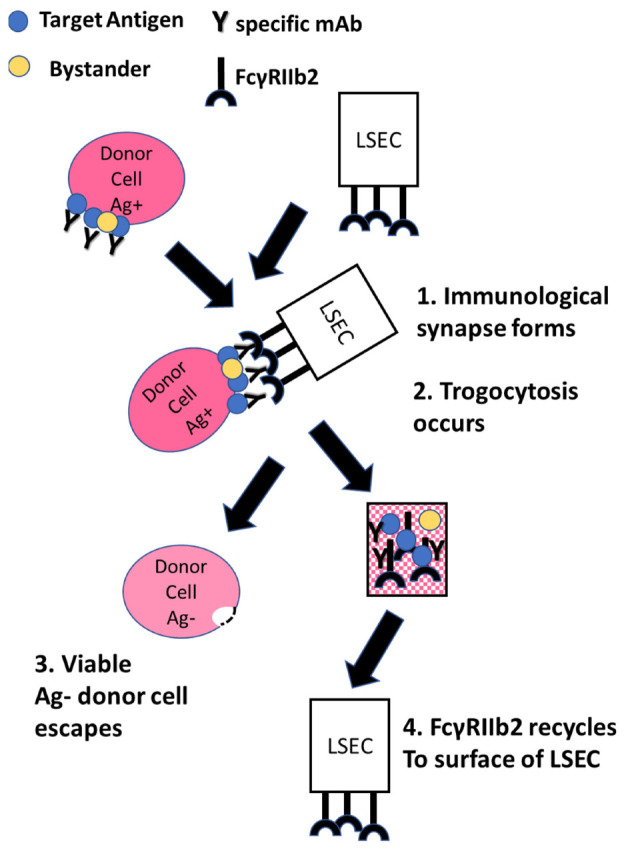
Schematic representation of FcγRIIb2-mediated trogocytosis. 1. A circulating IgG-opsonized target cell encounters LSEC. 2. An immunological synapse forms between the cell-bound immune complex and FcγRIIb2 on the surface of the LSEC. 3. The immune complex, cell membrane, and some bystander proteins located in proximity to the target antigen are transferred from the target cell to the LSEC and internalized. The target cell escapes with loss of varying amounts of immune complexes and loss of some membrane. 4. FcγRIIb2 recycles to the surface of the LSEC, ready to repeat the cycle.

Figure 1 schematically depicts our hypothesized mechanism of FcγRIIb2-mediated trogocytosis, as follows: 1, IgG-opsonized target cells circulate through the liver and form an immunologic synapse with acceptor cells, in particular LSEC, which express the inhibitory receptor FcγRIIb2; 2, this receptor mediates rapid trogocytosis of the donor cell-bound IgG immune complexes into the LSEC without destruction of the donor cells; 3, the antigen-negative target cells escape; 4, FcγRIIb2 recycles to the surface of the LSEC. This general process has also been described as “antigenic modulation” and/or antibody mediated immune suppression [34,35,36]; we suggest, based on reports that we cite below, that essentially the same reaction occurs in all these cases. A key part of the process appears to also include the transfer to the acceptor cell of membrane fragments from the donor cell that were in close contact with the IgG-opsonized donor cell-targeted antigen [37,38]. Indeed, membrane proteins in close contact with the acceptor cell are also transferred to the acceptor cell (“innocent bystander transfer”) [37,39,40]. Whether trogocytosis requires the action of one or more proteases has still not been resolved [37,41,42]. Our discussion is focused on FcγRIIb2-mediated trogocytosis, but this same pathway is also followed by activating Fcγ receptors on macrophages and neutrophils [37,43,44,45].

**Figure 2:**
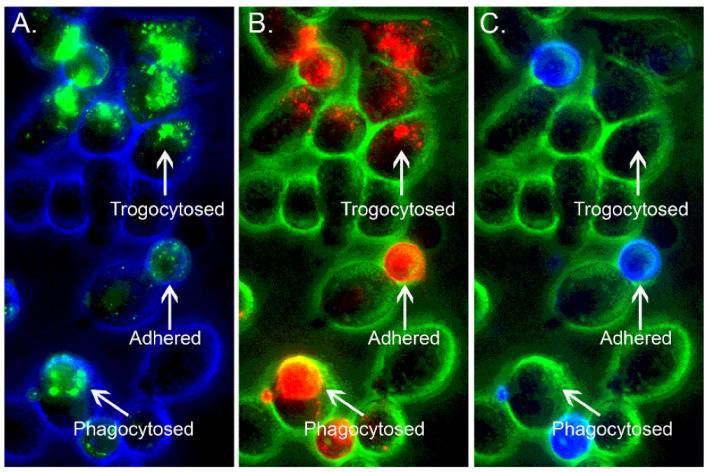
Phagocytosis and trogocytosis are demonstrable in the same timeframe. Mouse 38C13 B cells expressing human CD20 were dyed with PKH26 ((**B**), red) and then opsonized with Al488 rituximab ((**A**), green). The cells were then reacted with adhered J774 macrophages for 30 min and finally stained with Al647 anti-mouse IgM ((**C**), blue). Based on the staining patterns, it is possible to identify adhered cells (blue, red, and green), phagocytosed cells (red and green, but no blue staining), and portions of trogocytosed cells (fragments of only red and green) [38,54]. Adapted with permission from Ref. [38], 2010, The American Association of Immunologists, Inc.

An extensive literature, based in part on in vitro investigations with macrophages, describes the removal of circulating IgG-opsonized cells from the bloodstream by liver and splenic macrophages via phagocytosis [18,46,47,48,49,50,51]. Therefore, the immune system must “decide” between phagocytosis (the entire IgG-opsonized cell is removed) and trogocytosis (only immune complexes and membrane fragments are removed). How this differentiation between the two FcγR-mediated processes is adjudicated remains an interesting and important question. Indeed, the fate of the opsonized cell and associated IgG appears to depend on many factors, including the following: the IgG density and distribution on the opsonized cell; which acceptor cells are exposed to the immune complexes; and what the FcγR status and phagocytic capability is on the potential acceptor cell. We will cite certain instances in which there is good evidence, in vitro and in vivo, that under certain conditions both phagocytosis and trogocytosis can occur simultaneously [52,53]. One example of this phenomenon is illustrated in Figure 2.

## 3. Phagocytosis and Trogocytosis Occur in Patients with CLL Treated with Anti-CD20 mAbs 

Our clinical studies of the effects of the usual standard high doses of CD20 IgG mAbs rituximab (RTX) and ofatumumab (OFA) in the treatment of chronic lymphocytic leukemia (CLL) revealed that the CD20 mAbs rapidly bind to circulating CLL cells which are then cleared from the bloodstream most likely due to the phagocytic action of macrophages in the liver and spleen [19]. This phagocytic cell-based mechanism for extravascular clearance of circulating IgG-opsonized cells was first described by Frank’s laboratory more than 50 years ago [48,49,50,51]. It has now been validated for rituximab based on several elegant real-time intravital imaging studies in mouse models reported by Montalvao and Gul [55,56,57] and also based on the more recently described in vitro studies of RTX-mediated phagocytosis reported by Zent and colleagues [58,59,60]. 

We found that very early during the infusions, not only were CD20-positive CLL cells rapidly cleared, but at the same time the levels of CD20 on CLL cells remaining in the circulation also decreased precipitously in ~15 min, even after infusion of only 10–30 mg of the CD20 mAb [52,53]. Although it could be argued that this indicated that the cells with the most CD20 were cleared first, we also found that these remaining cells had levels of CD20 that were considerably lower than the starting CD20 levels before RTX or OFA infusion. We therefore suggest that, simultaneously with phagocytosis of some circulating opsonized cells, substantial amounts of the cell-bound immune complexes (CD20 and RTX/OFA) were “shaved” (trogocytosed) [37] from opsonized cells due to the action of FcγRIIb2 on LSEC. We also note that trogocytosis has been used as a qualifying criterion for certifying RTX “Biosimilars” [61].

Later, as the CD20 mAb infusions continued, additional CLL cells re-equilibrated into the bloodstream from other depots. Even though there were still ample amounts of circulating CD20 mAb, these cells were not cleared and in fact they had very low levels of CD20. It is likely that under these later conditions the phagocytic capacities of liver and splenic macrophages were temporarily saturated, but trogocytic removal of the CD20-IgG complexes bound to the re-equilibrated CLL cells continued. The confirmatory evidence for this scenario is based on the following key observation: The remaining circulating CLL cells were tagged with substantial amounts of covalently bound C3d [52,53]. This would indicate that RTX/OFA had bound to CD20 on the cells and activated complement, thus opsonizing them with C3b which soon decayed to C3d. However, the cells were not cleared from the circulation by phagocytosis, most likely due to temporary macrophage exhaustion. Instead, trogocytosis (and removal of CD20 and bound RTX/OFA) continued until plasma levels of RTX/OFA declined sufficiently. 

Several studies have demonstrated that macrophage exhaustion in CD20 mAb therapy for CLL is only temporary. After the liver and splenic macrophages have processed and digested their phagocytosed cargo (RTX- or OFA-opsonized CLL cells), they will then be “ready” (within ~48 h) to clear additional cells [53,60]. On this basis, we have suggested and provided evidence that frequent low doses of CD20 mAbs (~30 mg, 3 times per week) would mitigate trogocytosis and be far more effective for CLL treatment [53]. Lower doses of mAb result in the faster recovery of CD20 levels on circulating cells and make additional mAb infusions more effective. Recent clinical studies reported by the Zent group have further validated this approach [62,63]. 

Based on these considerations, it is not surprising that trogocytosis has also been reported for RTX therapy that did not target cancer cells. Crickx et al. found that when patients with immune thrombocytopenia received RTX infusions, RTX-resistant memory B cells were produced [39]. These cells had undetectable levels of CD20 and there was clear evidence for “innocent bystander” removal of associated markers CD19, CD21, CD22, and CD79b.

## 4. Antibody Mediated Immunosuppression/Antigenic Modulation on E

Research on blood transfusions is now centered on the minor E antigens (e.g., KEL) and on understanding the mechanism by which infused polyclonal anti-RhD (rhesus antigen) antibodies suppress immune responses to RhD in RhD-negative mothers who give birth to children whose E are positive for RhD [64]. An investigation in an RhD-positive individual who received anti-RhD treatment revealed that the anti-RhD antibodies promoted substantial loss of RhD on circulating E [65]. This finding is most readily explained by trogocytosis mediated by the anti-RhD antibodies.

In a mouse model reported by Liu et al., anti-KEL antibodies were first infused, followed by engineered KEL-positive mouse E. The cognate anti-E antibody was found to downregulate levels of KEL [66]. The Lazarus group concluded that the most likely mechanism for epitope masking induced by antibodies was trogocytosis [35]. Both groups have noted that in the studies of Liu et al. “There was clearance of roughly 50% of the transfused RBCs. In the cells that remained in the circulation, there was virtually no detectable KEL glycoprotein present on the erythrocytes as assessed by flow cytometry and Western blotting” [35,66]. We wish to emphasize that these results very closely recapitulate our findings (including Western blots) when CLL patients receive RTX or OFA infusions: there is (initially) clearance of more than half of the CLL cells and the remaining circulating cells express very low levels of CD20 [67]. 

These findings were extended by Maier et al. in a separate but similar mouse model in which two antigens, KEL and hen egg lysozyme peptide (HEL), were expressed on mouse E that were then infused, followed by antibodies specific for KEL and/or HEL [34]. These experiments demonstrated that specific antibody-mediated loss of one of the antigens does not induce removal of the other antigen; we suggest that this pattern can be variable. In the CLL/RTX system, there is indeed some “innocent bystander” loss of unrelated antigen CD19 when CD20 is removed by trogocytosis [37,40]. We suggest that future studies of these phenomena will provide information on the nearest neighbor interactions. For example, we have reported that CD20 and CD37 are very closely colocalized on CLL cells [68] and based on this observation, it is likely that if one of these proteins is removed from a CLL cell by direct trogocytosis, then levels of the other protein will likely decrease as well. 

Maier et al. also explored whether macrophages played a direct role in modulating either the KEL or HEL antigens. They found that clodronate treatment, which destroys macrophages, had no effect on anti-HEL induced downregulation of HEL, thereby suggesting that the HEL antigen was removed by a mechanism that was independent of macrophages. However, they found that clodronate partially inhibited the anti-KEL mediated suppression of KEL on E, suggesting that macrophages in the liver may have removed some of the KEL/anti-KEL immune complexes [34]. We note that in this later case, the suppression was only partial. It is therefore likely that the “smoking gun” for partial removal of both KEL and full removal of HEL resides in the action of LSEC. This possibility can be tested in mice in which FcγRIIb2 is knocked out, or by blocking the receptor with a specific antibody [22,69]. We also suggest that immunohistochemical interrogation of isolated Kupffer cells and LSEC for the targeted antigens should be most informative. 

The report of Elayeb et al. is also quite relevant [70]. They examined the potential of anti-mouse CD20 antibody therapy to prevent E alloimmunization in a mouse model. They discovered that marginal zone B cells were not destroyed by the anti-CD20 mAb (presumably they were not in direct contact with macrophages), but instead they were subject to trogocytosis, thus leading to substantial decreases in their expression of CD20. This observation is important because it is not based on a cancer model and macrophage “exhaustion” is not an issue. The identity of the effector cells that mediated trogocytosis of the marginal zone B cells remains to be determined. 

The effect of daratumumab (DARA)-mediated trogocytosis in reducing levels of CD38 in targeted MM cells is reviewed below. However, CD38 is also expressed on E and recently Sullivan et al. reported results from a clinical study of DARA which demonstrated that infusion of DARA also induced loss of CD38 from E with no evidence of hemolysis [71]. It is very likely that this loss is mediated by trogocytosis and we suggest that in vitro studies with human E, DARA, and acceptor cells should follow the same patterns we have described earlier in this section. 

## 5. NK Cells Can Act as Donors or Acceptors in Trogocytosis

The group of Lopez-Botet recently reported an in vitro investigation that evaluated the potential of NK cells to destroy B cells that were infected with Epstein–Barr virus [72]. They found that in the presence of serum from EBV-infected individuals (containing anti-EBV antibodies) NK cells removed viral particles (VP) from the B cells and eventually internalized the particles. The transfer process did not occur in the absence of EBV-positive serum, thus emphasizing that it was the immune complexes on the B cells that were removed. In addition, CR2 (CD21), the binding site for VP, was also transferred to the NK cells, along with some PKH26, a membrane dye used to label the B cells. Evidence for the “innocent bystander” transfer was also pronounced: both CD20 and CD19 (closely associated with CD21) were found on the NK cells. We suggest that this experiment can also be replicated in the absence of anti-EBV serum if a suitable mAb specific for VP bound to the B cells is used to mediate opsonization. Alternatively, if NK cells are to be useful in the therapy of EBV infection, strategies that block trogocytosis will be needed. 

The group of Childs investigated the failure of an NK cell-specific checkpoint inhibitor, IgG4 mAb IPH2101, specific for KIR2D on NK cells, to achieve clinical efficacy in a trial for the treatment of MM [73]. Their previous in vitro investigations indicated that blocking KIR2D with this mAb enhanced NK cell-mediated killing of MM cells. However, the lack of efficacy in vivo during the clinical trial was, in retrospect, readily understood based on their observation of a substantial reduction in the levels of KIR2D on patients’ circulating NK cells that were analyzed after treatment with mAb IPH2101. These clinical observations were reinforced and validated based on their subsequent in vitro experiments, which demonstrated that monocytes and neutrophils could mediate trogocytosis of the KIR2D-IPH2101 immune complex from NK cells. They found that trogocytosis could be blocked in vitro in the presence of IVIG, which presumably inhibited FcγR. Whether IVIG can be used therapeutically to block trogocytosis but still allow other therapeutic mAb functions to remain intact is yet to be determined. This study is also important because it demonstrates that even IgG4 can mediate trogocytosis; IgG4 has often been selected for certain therapeutic applications including antibody drug conjugates because it is presumed to weakly interact with FcγR and complement [74]. However, the study of Childs clearly demonstrates that immune complexes containing IgG4 are indeed subject to trogocytosis, and as reviewed below, this may explain the liver damage associated with certain antibody-drug conjugates (ADC) prepared with IgG4.

## 6. Trogocytosis in HIV Disease

Richardson et al. have developed an elegant model system to evaluate the role of HIV-specific antibodies in mediating trogocytosis [75]. The method is based on loading monomeric or trimeric gp120 on a CD4-positive T cell line followed by opsonization with polyclonal anti-HIV antibodies isolated from the sera of HIV-infected patients. The investigators found that membrane dyes, as well as gp 120, CD4, and HIV-specific IgG on the substrate CD4+ cells, are indeed transferred to acceptor THP-1 cells in a rapid reaction (1 hr or less). As expected, in the absence of anti-HIV IgG antibodies, there was negligible transfer of the other components to the acceptor THP-1 cells. Moreover, the selected blockade of FcγRIIa or FcγRIIb almost completely abrogated trogocytosis. This is an important finding because it provides a clear instance in which the inhibitory receptor FcγRIIb2 mediates trogocytosis. There was a moderate loss of viability in the donor gp120-primed T cells subject to trogocytosis, and whether trogocytosis therefore may serve an immunotherapeutic role with broadly neutralizing anti-HIV mAbs to target infected T cells is likely to be the subject of future investigations.

## 7. Daratumumab Mediates Trogocytosis of CD38 on Numerous Cells

The mAb DARA, specific for CD38, has proven to be moderately successful in the treatment of MM based on targeting and clearing CD38-positive MM cells [76]. However, in analogy to the action of CD20 mAbs RTX and OFA, DARA also down-regulates the expression of CD38 on targeted MM cells that are not cleared [77,78]. This process has been demonstrated to be due to trogocytosis and we note that in analogy to our “timed” approach for CD20 mAb infusions, Nijhof et al. [79] recognize that waiting to re-infuse patients with additional doses of DARA should “provide the rationale for retreatment with daratumumab after sufficient time to allow CD38 expression levels to return to baseline on remaining MM cells”.

The results of experiments conducted by Krejcik et al. on blood samples taken from patients treated with DARA revealed that not only does DARA induce rapid reduction in CD38 on MM cells, it also reduces CD38 levels on NK cells, T cells, and B cells [77]. In vitro experiments with a CD38-positive cell line demonstrated that effector cells (monocytes and granulocytes) were required to mediate the loss of CD38 induced by DARA. The loss of CD38 could be partially blocked in vitro upon addition of excess human IgG, thus identifying FcγR on these cells as likely playing a key role in the reaction. These investigations also eliminated internalization by the opsonized cells as a mechanism for loss of CD38. Moreover, along with the loss of CD38, there was “innocent bystander” reduction in levels of other cell-surface proteins, including CD56, which is presumably located close to the CD38 molecules that are removed from the cells. Experiments with membrane dye-labeled donor cells revealed that membrane fragments are also transferred from the donor cells to acceptor cells. These findings closely recapitulate our observations of trogocytosis mediated by RTX and OFA [37,52] and again speak to the generality of the trogocytic mechanism.

## 8. Antibody Drug Conjugates (ADC) 

Although unconjugated immunotherapeutic mAbs have demonstrated considerable success in a variety of clinical applications, in many cases their efficacy is limited. Therefore, there are now considerable efforts to enhance the cytotoxic action of mAbs in the form of antibody-drug conjugates (ADC) [80,81,82]. These agents are composed of a mAb that is covalently coupled to a cytotoxic payload/poison that should allow targeting to and killing of specific (malignant) cells. Under optimum conditions, the agent should bind to the targeted cell and then, upon its internalization, release the cytotoxic agent and thus kill the cell. As has been reviewed, therapies with an ADC can induce toxicity and injury to normal tissue and cells via a number of specific and non-specific pathways [81]. Our interest in this issue centers on the use of ADC to target and destroy circulating cells. If this is the case, then we would predict that some fraction of the ADC-opsonized target cells would deliver the ADC to LSEC for trogocytosis, and the acceptor LSEC would likely be killed after they internalized the ADC. 

The ADC immunotherapeutic cell-killing paradigm has been clearly demonstrated for an ADC that targets CD33 on myeloid cells, Mylotarg (gemtuzumab ozogamicin) [74,83]. It has been used successfully in the treatment of acute myeloid leukemia (AML) based on its ability to bind to and destroy CD33-positive AML blast cells. Although Mylotarg also eliminates CD33-positive normal cells, its principal and most serious adverse effect is liver toxicity, in particular, sinusoidal obstruction syndrome (SOS) that appears to be a consequence of damage to LSEC [84]. Whether this toxicity is due to trogocytosis or due to targeting of CD33-positive cells in the liver has not been established. Tests of a “non-binding” Mylotarg analogue in cynomolgus monkeys gave rise to similar damage to LSEC and this finding would rule out target-specific trogocytosis, if indeed the analogue was not bound to circulating cells [85]. Mylotarg was at one time withdrawn from the market due to liver toxicity, but it was later re-approved as a consequence of a trial in which “lower, fractionated doses” were found to reduce liver damage [86]. As noted above, we and others have advocated the use of lower frequent doses of CD20 mAbs to minimize trogocytosis, and the paradigm now used for Mylotarg may have similar action.

It is interesting that earlier, van der Velden and colleagues reported that the binding of Mylotarg to cells can induce downregulation of CD33 in the presence of acceptor monocytes [83]. On this basis, it would seem reasonable to react Mylotarg-opsonized cells with potential acceptor cells (LSEC or THP-1 cells) to determine if the ADC bound to cells is susceptible to trogocytosis. This approach can be easily extended to examine other ADC that might induce liver toxicity, and it would seem reasonable to include this test in an initial screening of a newly developed potential ADC. 

The studies of McDonald et al. with an ADC that targets CD22 (inotuzamab ozogamicin) are also noteworthy [87]. They examined the use of this ADC in the treatment of hematologic malignancies and found a much lower incidence of SOS with this agent than with Mylotarg, but reported that it appeared to promote liver injury in 7.9% of the treated patients. These investigators indeed considered the possibility that “endocytosis of ADCs by the FcγRIIb2 receptor on LSEC is the proximate cause of LSEC damage” because there are no CD22-positive cells in normal liver tissue. We agree with this proposed mechanism except we propose that the transfer of the ADCs to LSEC is accomplished much more efficiently if the ADCs are bound to circulating targeted cells in multivalent immune complexes, thus allowing for more effective trogocytic transfer to the LSECs.

## 9. Variations in the Outcome of FcγR-Mediated Transfer Reactions including Results with Epidermal Growth Factor Receptor (EGFR, HER2) 

In the examples of trogocytosis that we have cited, the substrate donor cancer cells escape alive from the effector cells after parting with immune complexes (Figure 1). This escape pattern is not followed when breast cancer cells are opsonized with an anti-HER2 mAbs such as trastuzumab (TRA) [43,45]. TRA has been used successfully in the treatment of HER2-positive breast cancer and several mechanisms of action for the efficacy of this mAb have been postulated, including ADCC and phagocytosis mediated by effector cells [88]. Susuki et al. reported that monocytes and NK cells can mediate trogocytosis of TRA-opsonized cells and suggested that this could be part of the therapeutic action of TRA [89]. 

The groups of Ward and van den Berg have demonstrated that macrophages and neutrophils, respectively, also mediate trogocytosis of the TRA/HER2 complexes bound to breast cancer cells, but in these cases the substrate cells are killed [43,45]. Macrophage-mediated killing and trogocytosis is slow and requires several days, but neutrophil-mediated trogocytosis is evident within less than one hour and is clearly accompanied by the transfer of plasma membrane and TRA to the neutrophils. Trogocytosis leading to cell death has been termed trogoptosis [43]. There are several possible explanations for this dramatic exception: it is likely that the plasma membranes of the breast cancer cells suffer excessive damage when the immune complexes are removed by trogocytosis, because inherent in the reaction is the removal of sections of the plasma membrane of the cells associated with the target antigen. There can be ~1–2 million HER2 proteins on breast cancer cells, while the number of CD20 molecules on B cells varies between 20,000–250,000/cell [88,90,91]. It is certainly reasonable to expect that removing 2 million HER2 proteins from the plasma membrane of a breast cancer cell is far more likely to be a fatal and disruptive membrane insult than if only 20,000 CD20 proteins are excised from a CLL cell. Alternatively, if the HER2 protein is critical for normal cell function, then it is possible that the cell can “not live without it!”. The HER2 target follows otherwise similar patterns of trogocytosis, but because the cell is killed it is a singular and important exception in the patterns we have described; it will be interesting to identify and characterize other future cellular mAb targets in which trogocytosis is a lethal event. 

Along these lines, van den Berg’s group has extended their studies to *include* neutrophil-mediated killing of CD20 antibody-opsonized B cells [92]. They found that by blocking the CD47/signal-regulatory protein α (CD47-SIRPα) interaction (the “don’t eat me” signal) in the presence of sodium stibogluconate (which can block tyrosine phosphatase SHP-1), it was possible to use neutrophils to kill CD20-opsonized cells. The details of this dramatic “reversal of fortune” mechanism are still under investigation, but it is exciting to recognize that even for CD20 targets, it may be possible to use trogocytosis to kill targeted cells instead of helping them to escape.

In a related observation, Vijayarghavan et al. developed and evaluated the activity of amivantamab, a bispecific mAb construct that targets EGFR and cMet. They found that in the presence of monocytes or macrophages the bispecific mAb mediated killing of lung cancer cell lines, and the principal cytotoxic mechanism was trogocytosis, which resulted in the downregulation of both EGFR and cMet [93].

There are instances in which liver macrophages appeared to rapidly remove mAbs from opsonized cells; Liew et al. reported that Kupffer cells “ripped large fragments off crawling antibody-coated iNKT cells”, which resulted in the killing of the cells, and the investigators defined this process as antibody-dependent fragmentation [94], which it would also appear to fit with van den Berg’s definition of trogoptosis. Arlauckas et al. reported in vivo imaging studies of a macrophage-dependent tumor-resistant pathway in which mAbs specific for PD-1 bound to tumor-infiltrating CD8 + T cells were rapidly removed from the cells by macrophages. However, in vitro experiments indicated there was no evidence for the transfer of membrane fragments or PD-1 antigen from these donor cells to macrophages, thus precluding the strict identification of this process as trogocytosis [95]. Additional experiments are required to resolve these observed differences. 

## 10. The Case for FcγRIIb2

Our in vitro investigations along with those of Golay, Ward, and van den Berg indicate that effector cells that express FcγR, especially macrophages and neutrophils, all appear to be able to mediate trogocytosis [37,43,44,45]. Most of these studies focused on the activating receptors, but recently Leusen’s group has demonstrated that the inhibitory receptor, FcγRIIb2, can also mediate trogocytosis in a mouse model [96]. Anderson and colleagues have reported a substantial body of evidence that indicates that FcγRIIb2 (the only FcγR on LSEC) is capable of mediating very efficient and rapid clearance and internalization of small immune complexes and particles [21,23,26]. This is followed by rapid recycling of the receptor back to the cell surface, thus allowing for very effective and sustained trogocytosis. Datta-Mannan examined the clearance kinetics of bispecific antibody complexes that are rapidly cleared from the bloodstream like small immune complexes [25]. They found that treatment with clodronate (thus killing macrophages) had little effect on the removal of these substrates in a cynomolgus monkey model. This again emphasizes the likely importance of LSEC in the trogocytic process.

Moreover, as we noted earlier, there appears to be two separate fates for cell-bound immune complexes when RTX or OFA is first infused into the circulation of a patient with CLL. A fraction of the opsonized CLL B cells are rapidly removed and we suggest they have come into contact with and are taken up by liver macrophages. This process cannot be 100% effective based on a single pass of high concentrations of RTX-opsonized cells through the liver, and in addition there is compelling in vitro evidence indicating that this process can be temporarily saturated [58,59,60]. We suggest that some of the cells not immediately taken up by macrophages are instead processed and trogocytosed by LSEC. The capacity of LSEC to interact with, process, and internalize immune complexed substrates is substantial. Bhandari et al. [31] note that within the liver there are 2.5 times more LSEC than Kupffer cells, which clearly emphasizes the potential of these cells to clear immune complex substrates. One of the other important properties of LSEC is that based on the action of FcγRIIb2, they can dispose of potentially harmful immune complexes without inflammation [27]. Scholzel et al. reported that LSEC are capable of mediating trogocytosis [24]. They found that the LSEC acquired MHC-1 antigens from hepatic stellate cells in a process that enhances immune surveillance. We suggest that by combining trogocytosis with clearance of small immune complexes, LSEC would appear to play a unique role in the non-destructive processing of IgG-antibody opsonized cells.

It also should be noted that the LSEC only “nibble” and do not process entire cells, and therefore it is likely that their trogocytic capacity is not easily saturated. Indeed, our clinical results, based on the treatment of CLL patients with RTX or OFA, can best be interpreted as representing the saturation of phagocytic mechanisms but continuation of trogocytosis because large numbers of circulating cells with substantially reduced levels of CD20 are most readily demonstrable [52,67]. We again emphasize that this observation is recapitulated in the mouse model studies, which investigated the targeting of the RhD and KEL antigens [34,64,65].

The hypothesis that we have set forth can be tested in many ways. First, experiments in mouse models can be conducted in which mice with FcγRIIb2 knocked out can be compared to studies with wild type mice [22]. Alternatively, experiments with mAbs that block FcγRIIb2 can be conducted [69]. Other approaches include blocking FcγRIIb2 with either IVIG or Fc multimers [97,98]. Additionally, the introduction of clodronate in either mouse or monkey models would be expected to have minimal effects on the clearance of the substrates, as demonstrated in the cited studies of Datta-Mannan and Maier et al. [25,34]. Moreover, as we noted previously, microscopy and histochemical analyses can also be used to specifically identify the sites in which the cleared complexes are deposited. We emphasize that these experiments are not limited to tests with CD20 mAbs. We have described several different systems, almost all of which appear to undergo trogocytosis and therefore should be amenable for these tests.

## 11. Back to the Future: Re-Evaluation of the Immune Adherence Phenomenon/Trogocytosis in a Mouse Model

It is important to note that only primate E express CR1. The late Professor Robert Finberg and colleagues addressed this issue by developing genetically engineered mice that express human CR1 on their E [99]. The use of these mice will allow for a rigorous re-examination of Nelson’s immune adherence phenomenon. Experiments in which immune complexes are bound to these E can be conducted, based on using mouse mAbs specific for CR1 as surrogates for human C3b [18,100]. For example, the immune complexes can first be bound to E ex vivo and then infused into mice, or they can be bound to E in vivo. In each case, it is our prediction that CR1 and the immune complexes will be taken up by LSEC. Tests to block this reaction with IVIG, or with mAbs that block FcγRIIb2, are all feasible and should be quite informative.

## 12. Other Possible Mechanisms

The Southampton group has reported that CD20, along with cell-bound CD20 mAbs, can be directly internalized by B cells, and that this reaction is mediated in part by FcγRIIb on the B cell. This process will of course mitigate CD20 mAb-based therapies, and on this basis, it is reasonable to develop mAbs that inhibit the action of this receptor in order to block the internalization reaction [69,101,102]. Efforts to bring such mAbs to the clinic for treatment of certain cancers are underway [102].

However, we have already reported that internalization is much slower than trogocytosis: internalization requires ~6 h, while trogocytosis is complete in less than 30 min [103]. This dichotomy between internalization and trogocytosis with respect to CD20 mAbs in particular was reviewed by Stevenson [104]. We have emphasized that there is a clear generality and repeating pattern displayed in all the very well-documented FcγR-mediated trogocytic processes that have been reported, many of which are reviewed herein. These reactions are all rapid; they require IgG mAbs that specifically react with a considerable diversity of cell-based antigens besides CD20, and the cellular substrates include, in addition to B cells, E, NK cells, T cells, and MM cells. Moreover, we have cited substantial evidence that FcγRIIb2 on LSEC plays a key role in trogocytosis, and therefore the use of mAbs that block this receptor may prove to be particularly useful in safely suppressing trogocytosis [102].

## 13. Summary and Future Directions

There has been considerable progress in our understanding of trogocytosis since Joly and Hudrisier first inquired about the meaning and purpose of this reaction almost 20 years ago [1]. The transfer of membrane markers from donor cells to acceptor cells can “fool” the immune system and change immune effector cells to substrates for unexpected reactions. We have cited the increasing evidence that trogocytosis of cell-bound IgG-opsonized substrates mediated by FcγR on a variety of cells appears to follow a general pathway first enunciated by Nelson in the 1950s. Based on the similar reaction patterns manifested in these processes, we have hypothesized that FcγRIIb2 plays a major role in these reactions. Moreover, tests for targeted inhibition of these forms of trogocytosis by focusing on blocking this receptor can allow for its rigorous testing. The elucidation of the details of the “path taken” by FcγR-mediated trogocytosis has the potential to allow for the development of novel therapies that can potentially block or enhance this reaction, depending upon whether the process leads to unfavorable or positive biological effects.

## Data Availability

Not applicable.

## Abbreviations

ADC	antibody-drug conjugate
CLL	chronic lymphocytic leukemia
CR1	complement receptor 1, CD35
DARA	daratumumab, specific for CD38
E	erythrocyte
EBV	Epstein–Barr virus
FcγR	Fcγ receptor
HEL	hen egg lysozyme peptide
LSEC	liver sinusoidal endothelial cells
MM	multiple myeloma
OFA	ofatumumab
RTX	rituximab
TRA	trastuzumab
VP	viral particle
